# Associations of age and sex with brain volumes and asymmetry in 2–5-week-old infants

**DOI:** 10.1007/s00429-018-1787-x

**Published:** 2018-11-02

**Authors:** S. J. Lehtola, J. J. Tuulari, L. Karlsson, R. Parkkola, H. Merisaari, J. Saunavaara, T. Lähdesmäki, N. M. Scheinin, H. Karlsson

**Affiliations:** 1 FinnBrain Birth Cohort Study, Turku Brain and Mind Center, Institute of Clinical Medicine, University of Turku, Lemminkäisenkatu 3, 2nd floor, 20520 Turku, Finland; 2 Department of Child Psychiatry, University of Turku and Turku University Hospital, Turku, Finland; 3 Department of Radiology, University of Turku and Turku University Hospital, Turku, Finland; 4 Department of Future Technologies, University of Turku, Turku, Finland; 5 Department of Medical Physics, Turku University Hospital, Turku, Finland; 6 Department of Pediatric Neurology, University of Turku and Turku University Hospital, Turku, Finland; 7 Department of Psychiatry, University of Turku, Turku University Hospital, Turku, Finland

**Keywords:** Brain asymmetry, Infant brain, MRI, Brain volume, Infant age, Sexual dimorphism

## Abstract

Information on normal brain structure and development facilitates the recognition of abnormal developmental trajectories and thus needs to be studied in more detail. We imaged 68 healthy infants aged 2–5 weeks with high-resolution structural MRI (magnetic resonance imaging) and investigated hemispheric asymmetry as well as the associations of various total and lobar brain volumes with infant age and sex. We found similar hemispheric asymmetry in both sexes, seen as larger volumes of the right temporal lobe, and of the left parietal and occipital lobes. The degree of asymmetry did not vary with age. Regardless of controlling for gestational age, gray and white matter had different age-related growth patterns. This is a reflection of gray matter growth being greater in the first years, while white matter growth extends into early adulthood. Sex-dependent differences were seen in gray matter as larger regional absolute volumes in males and as larger regional relative volumes in females. Our results are in line with previous studies and expand our understanding of infant brain development.

## Introduction

The development of the central nervous system (CNS) is a rapid and plastic process during the first years of life and is influenced by many intrinsic and extrinsic factors (Holland et al. 2014; Stiles and Jernigan 2010). During this sensitive developmental time period, an unfavorable environment can result in disruption of CNS growth, which may lead to alterations in brain structure or function that can manifest as pathological conditions later in life. Though the field of research on infant brain development is advancing rapidly, the ramifications of early structural variation are still under active investigation. The challenges of infant brain imaging studies are not only restricted to data acquisition and analysis but also its interpretation (Arthurs et al. 2012).

Despite the challenges of studying infant brain development, we argue that there are four main reasons for studying the topic more deeply. First, highly contradictory reports exist on the maturation process of the cerebral cortex, including both cortical thickening and thinning as a sign of maturation (Walhovd et al. 2016). Second, although previous studies have observed that longer gestation associates with larger infant brain volumes and, not surprisingly, with infant overall growth (Aanes et al. 2015; Bora et al. 2014; Dean et al. 2018; Haukvik et al. 2014; Holland et al. 2014; Knickmeyer et al. 2017; Mills and Tamnes 2014; Munakata et al. 2013; Peterson et al. 2000; Thompson et al. 2007; van Soelen et al. 2010; Østgård et al. 2014), most such studies compared preterm and term-born infants. Therefore, less is known about whether gestational length differences amongst term-born children influence differences in brain volumes. Third, sex differences are as yet understudied. A growing body of literature recognizes that male brains generally have larger intracranial volume (ICV) as well as gray and white matter volumes during development than female brains (Gilmore et al. 2007; Knickmeyer et al. 2008a, b, 2017; Thompson et al. 2007; Uematsu et al. 2012; van Soelen et al. 2010). In addition, male brains have been shown to grow faster than female brains during the first 3 months of life (Holland et al. 2014). Overall though, sex differences in infant brain volumes seem to be subtle, similarly to those seen in adults (Guo et al. 2016). Fourth, extant studies indicate that left–right asymmetries exist already in the fetal brain (Rajagopalan et al. 2011) and distinctive growth patterns can be seen in different parts of the neonatal brain (Dean et al. 2018; Deoni et al. 2011; Gilmore et al. 2007; Tanaka et al. 2013). However, some of the evidence is conflicting and thus the true developmental direction and location of these asymmetries and their relationships with sex and age are still unknown. Thus, further refinement of knowledge in this area is beneficial.

Overall then, as only few studies have been published on the effects of age and sex on neonate brain size and asymmetry, it is important to confirm these previous findings with replication and especially provide new information on the relationships between asymmetry and infant sex as well as age, as this interaction is a novel aspect in the field. Moreover, information on other important factors possibly influencing brain development, such as infant growth markers, is largely missing. Some connections between infant characteristics (lower birth weight and height and head circumference) and smaller brain volumes have been reported, but the strongest effects seem to arise within infant groups of restricted growth, for instance, preterm very-low-birth-weight or small for gestational age neonates (Aanes et al. 2015; Dean et al. 2018; Knickmeyer et al. 2017; Mills and Tamnes 2014; Østgård et al. 2014). The influences of these factors on the term-born brain still need to be defined.

In this study, we investigated the associations of age and sex with brain size as well as with brain asymmetry in 68 2–5-week-old infants. We hypothesized that age, calculated from either due date or birth date (both have been employed in previous literature), is associated with larger brain size and that males have larger brain volumes than females. As a novel approach, we describe the asymmetry patterns and interregional correlations among brain volumes and lobar asymmetry patterns in relation to age and sex. We hypothesized that brain asymmetry is similar in both sexes and is seen as rightward (i.e., the right side lobe being larger) in frontal and temporal lobes and leftward (i.e., the left side lobe being larger) in parietal and occipital lobes, and also that asymmetry is more prominent in older infants. Additionally, we explored if closely located brain regions have the greatest interlobar volume correlations and if infant growth markers associate with brain volumes.

## Methods

The study was conducted according to the Declaration of Helsinki, and was reviewed and approved by the Ethics Committee of the Hospital District of Southwest Finland.

### Participants

Participants were 68 healthy Finnish (Caucasian) neonates (31 males, 37 females) from 2 to 5 weeks of age (counted from the estimated due date). All the infants were born full-term [between gestational weeks (gwks) 37 and 42] and weighed more than 2500 g. The exclusion criteria for the infants were: occurrence of any perinatal complications with neurological consequences (e.g., hypoxia), scoring lower than 5 points in the 5 min Apgar, previously diagnosed CNS anomaly or an abnormal finding in a previous MRI scan. The families were contacted via telephone and eligibility for the study was assessed. After explaining the purpose and protocol of the study, a written informed consent was signed by the parents on behalf of each infant.

### Clinical characteristics

The descriptive information of the infant population is presented in Table 1. No sex differences were found regarding the duration of pregnancy; infant age at scan counted from the estimated due date or from the birth date; birth weight or birth length; head circumference; duration of the labor; maternal pre-pregnancy body-mass-index (BMI) or age at term, or paternal age at term; maternal smoking or antidepressive medication in the first or last trimester; or maternal education.

**Table 1:** Data descriptives

Variables	All subjects (*n* = 68), mean (SD)	Boys (*n* = 31), mean (SD)	Girls (*n* = 37), mean (SD)	*F*	*p*
Infant age at scan (from estimated due date) (d)	25.6 (8.1)	23.9 (7.6)	27.0 (8.3)	0.826	0.12
Infant age at scan (from birth date) (d)	25.5 (7.2)	25.3 (7.8)	25.2 (6.8)	2.33	0.928
Birthweight (g)	3564 (429)	3603 (403)	3531 (454)	0.526	0.494
Birth length (cm)	50.3 (1.7)	50.6 (1.7)	50.1 (1.7)	0.448	0.287
Head circumference (cm)	35.2 (1.3)	35.5 (1.2)	35 (1.3)	0.555	0.169
Duration of labor (min)	514 (293)	506 (291)	523 (299)	0.014	0.819
Maternal prepregnancy BMI (kg/m^2^)	24.3 (3.9)	23.4 (3.4)	25 (4.1)	1.267	0.095
Maternal age at term (y)	30.2 (4.5)	30.9 (4.3)	29.6 (4.6)	0.005	0.251
Paternal age at term (y)	31.6 (4.4)	30.8 (3.6)	32.2 (4.8)	1.423	0.279
Maternal education^a^	Number (%)	Number (%)	Number (%)	1.454^b^	0.483
(1) Elementary school	1 (1.5)	1 (3.2)	0 (0)		
(2) High school/career college	41 (62.1)	20 (64.5)	21 (60)		
(3) College/university	24 (36.4)	10 (32.3)	14 (40)		
Maternal smoking 1st trimester	7 (10.3)	3 (9.7)	4 (10.8)	0.023^b^	0.878
Maternal smoking 3rd trimester	3 (4.4)	1 (3.3)	2 (6.1)	0.258^b^	0.612
Maternal medication 1st trimester	5 (7.4)	4 (12.9)	1 (2.7)	2.576^b^	0.108
Maternal medication 3rd trimester	3 (4.4)	3 (10.3)	0 (0)	3.693^b^	0.055

Results of independent samples *t* tests: no significant differences between the sexes within the variables listed below

^a^Maternal medication = medication against depression/anxiety during pregnancy

^b^Note small subsample sizes

### Obstetric data and demographic information

Obstetric data were retrieved from the Finnish Medical Birth Register of the National Institute for Health and Welfare (http://www.thl.fi), and included length of pregnancy, gestational age at birth, head circumference, birthweight and birth length. Additional information was also collected as a part of the FinnBrain Study protocol, such as maternal pre-pregnancy body-mass-index (BMI) and maternal and paternal age.

### MRI acquisition

The participants underwent the MRI scans solely for research purposes and without clinical indications. The participants were imaged with a Siemens Magnetom Verio 3T scanner (Siemens Medical Solutions, Erlangen, Germany). The imaging protocol was 40 min long and included an axial PD-T2-TSE (Dual-Echo Turbo Spin Echo) sequence, a sagittal 3D-T1 (T1-weighted MPRAGE) sequence, and diffusion tensor imaging (DTI) sequence. Only T2-weighted images were used in the current study, as they provide a good contrast between the skull and the brain. The slice thickness of the PD-T2 sequence was only 1 mm, to achieve an isotropic voxel size of 1.0 mm^3^. TR (time repetition) time of 12,070 ms and effective TE (time echo) times of 13 ms and 102 ms were used to produce both PD-weighted and T2-weighted images from the same acquisition. The total number of slices was 128. Sequence parameters were optimized so that “whisper” gradient mode could be used in PD-T2 TSE and 3D-T1 sequences to reduce acoustic noise during the scan.

The scanning was performed at the Medical Imaging Centre of the Hospital District of Southwest Finland mostly during afternoon and evening hours though some scans occurred during the day on weekends. An experienced radiographer received the family, reviewed the imaging protocol with them, and confirmed the absence of any safety risks (inner ear implants, pacemakers or other metal devices or parts). The imaging was performed without anesthesia, during natural sleep, before which the infants were fed with milk (breast milk or formula) and swaddled into sleep in a vacuum mattress. The parents were able to be present in the scanning room during the whole scan and had the opportunity to quit the study at any point of the procedure. Both the parents and the infant were provided with sufficient hearing protection (ear plugs or wax and custom-sized ear muffs for all infants). The radiographer observed the scanning through a window with a microphone contact to a parent and a loudspeaker with which all the sounds from the scanning room were heard, which allowed the radiographer to know if the infant woke. A scanning session was ended if the baby did not fall asleep before or during the scan. After the scan, a small thank you gift was given to the family (a bop hat or body suit) for participation.

All the brain images were assessed by a pediatric neuroradiologist for any incidental findings and, if found to have one, the infant and the parents were given a chance for a follow-up by a pediatric neurologist. Developmental status has thereafter been normal for all of the participants, including those with incidental findings (the manuscript describing the incidental findings in greater detail has been submitted to a peer-reviewed journal), who were included in the study. The incidental findings were deemed not to affect brain volume estimates and have been found to be common and clinically insignificant in previous studies (Rooks et al. 2008; Whitby et al. 2004).

#### Image preprocessing

**Fig. 1:**
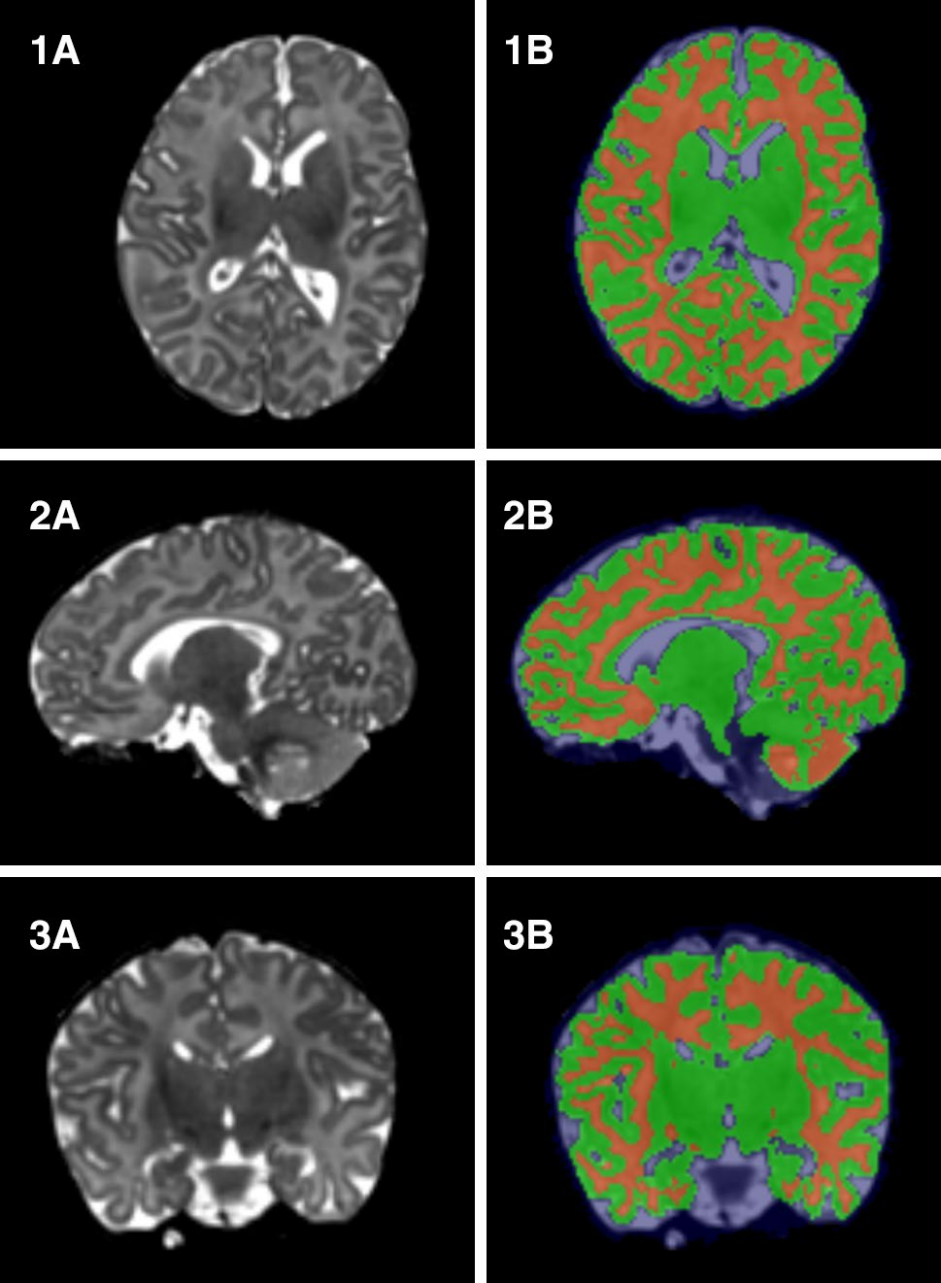
Representatives of (1) sagittal, (2) axial and (3) coronal views generated from a subject’s T2-weighted image. 1A–3A are the original T2-weighted images and 1B–3B are the segmented images, where the gray matter is marked with green and the white matter by red

The data were analyzed in iBEAT (Dai et al. 2013), an open source toolbox for processing infant brain images. After file conversion from dicom to analyze format, the images were preprocessed, which included reorientation and subject to N3 intensity non-uniformity correction. Then the skull was extracted with an iBEAT module and, if the extraction was incomplete, manual correction of the brain mask was done. Next, tissue segmentation, i.e., separation of gray and white matter [and cerebrospinal fluid (CSF)], was performed (Fig. 1). Finally, iBEAT labeled the different parts of the brain according to an infant-specific Automatic Anatomical Labeling (AAL) atlas. Total gray matter volume (TGM) was counted using the iBEAT segmented and AAL labeled data. While the cortical segmentation was performed successfully, the subcortical segmentation output contained parts of the white matter and was thus excluded from the analyses.

#### Brain volume calculation

Total brain volume (TBV) was calculated as a combination of all gray and white matter volumes, and intracranial volume (ICV) was calculated by adding cerebrospinal fluid (CSF) to TBV. Total cortical volume (TCV) was calculated by subtracting the subcortical parts from TGM. Lobar volumes were calculated using the AAL labeled data by combining together the volumes of separate anatomical brain parts belonging to a particular lobe. Ratios of the gray and white matter and lobar volumes in relation to total brain volumes, namely relative volumes, were also calculated for total white matter (TWM), TGM, TCV and lobar volumes so that TGM, TWM and TCV were expressed as relative to TBV and lobar volumes to TGM as they contained only gray matter.

### Statistical analyses

The IBM SPSS Statistics Version 23 for MAC was used for statistical analyses (Armonk, NY: IBM Corp.). The normality of the data was checked by visual confirmation and with Shapiro–Wilk test, and *p* values smaller than 0.05 were considered statistically significant, unless otherwise noted. To prevent possible Type I errors due to multiple tests, a Bonferroni correction was applied for all the analyses including multiple comparisons.

#### Associations with age and infant demographics

Infant age at scan varied if counted from the estimated due date or from the actual birth date, and thus, both ages were calculated and the associations between infant age and brain volumes were analyzed separately for both age variables using bivariate correlations. These results were then controlled for sex with partial correlation. The relationships between infant growth markers (head circumference, birth weight and length) and brain volumes were investigated with bivariate and partial correlations (controlling for sex and age).

#### Sex differences

To analyze differences in clinical variables between sex groups, a parametric test (*t* test) was used as the data was not found to deviate from normal distribution. Sex differences in mean brain volumes were examined with the independent samples *t* test. Simple linear regression, including age-by-sex interaction, was run to examine associations between gestational corrected age and brain volumes having sex as a fixed factor. All the analyses were repeated after excluding the infants (*N* = 5) exposed to maternal antidepressive medication during pregnancy and this did not affect the results.

#### Asymmetry

An asymmetry index (AI) (Dean et al. 2018; Tanaka et al. 2013; Uematsu et al. 2012) was calculated for each individual with the formula [(left − right / (left + right)) × 100]. Positive values represented leftward (left > right) and negative values rightward asymmetry. The statistical significance of hemispheric asymmetry was calculated using a one sample *t* test (testing against the reference value 0.00). If asymmetry was detected, differences between sexes were tested with independent samples *t* test. Simple linear regression was used to examine the relationship between asymmetry and age with sex as a covariate. Finally, bivariate correlations were implemented to detect possible correlations within regional brain volumes.

## Results

### Mean brain volumes

**Fig. 2:**
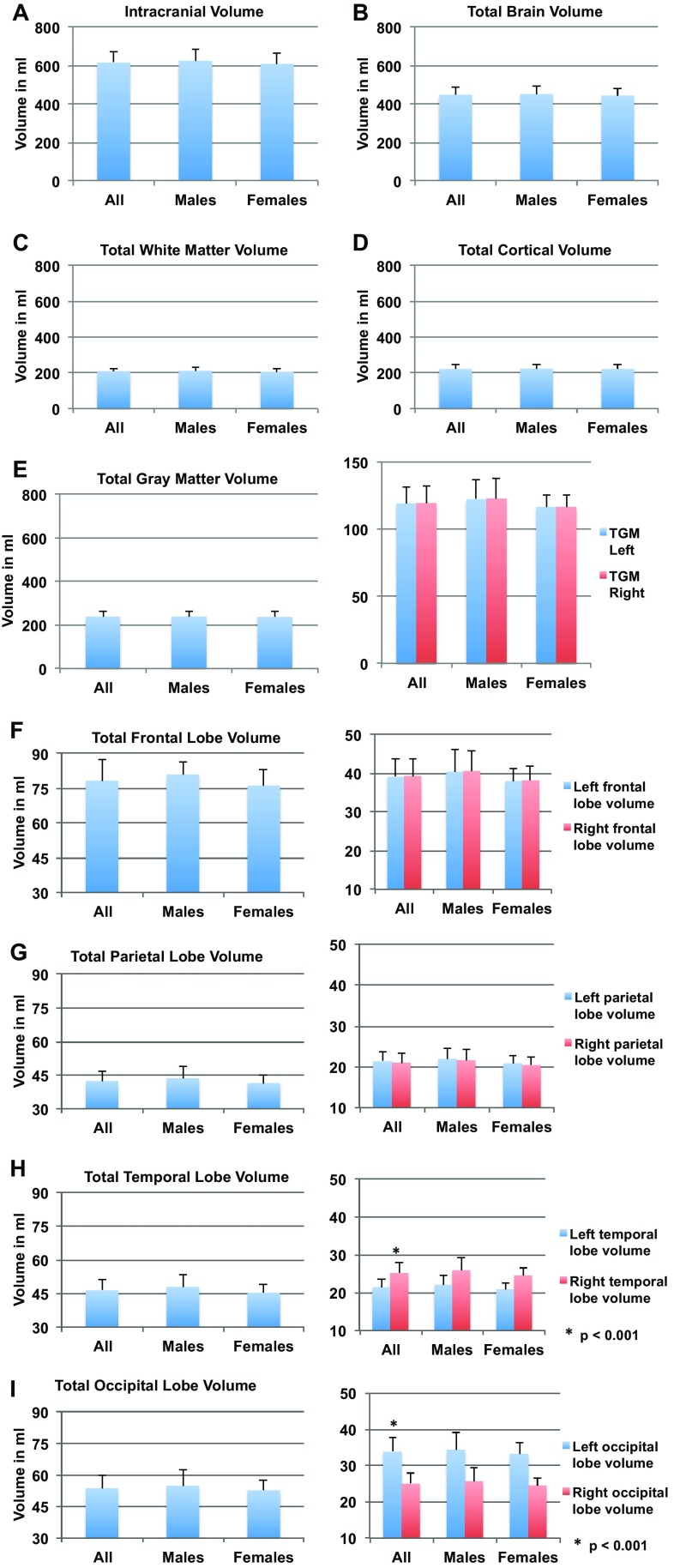
Mean total brain volumes (ml) in all subjects, males and females. **a** Mean intracranial volume. **b** Mean total brain volume. **c** Mean total white matter volume. **d** Mean total cortical volume. In the following, the left side represents the total volumes and the right side the hemispheric volumes in all subjects, males and females: **e** mean total gray matter volume, **f** frontal lobe volumes, **g** parietal lobe volumes, **h** temporal lobe volumes, **i** occipital lobe volumes. Statistically significant (*p* < 0.001, Bonferroni corrected for multiple comparisons) findings are marked by an asterisk and standard deviations by bars

The mean intracranial volume (ICV) for all subjects was 616 (SD 57.9) ml, the mean total brain volume (TBV) was 446 (SD 41.7) ml, the mean total gray matter volume (TGM) was 238 (SD 24.8) ml, the mean total white matter volume (TWM) was 208 (SD 18.4) ml and the mean total cortical volume (TCV) was 221 (SD 23.6) ml. The TGM volumes were highly similar in each hemisphere (119 cm^3^ SD 12.4) (Table 2; Fig. 2). The mean volume of the right side of the temporal lobe was greater than that of the left side (*p* < 0.001, Bonferroni corrected for multiple comparisons) and the volume of the left side of the occipital lobe was greater than that of the right side (*p* < 0.001, Bonferroni corrected for multiple comparisons) (Fig. 2).

**Table 2:** Mean brain volumes and the percentages relative to total volume

Cerebral structures	Mean volume in ml, (SD)	% of total volume^a^
TWM	208 (18.4)	46.6
TGM	238 (24.8)	53.4
Left	119 (12.4)	26.7
Right	119 (12.4)	26.7
TCV	221 (23.6)	49.6
Frontal lobe	78.3 (9.1)	17.5
Left	39 (4.6)	8.7
Right	39.2 (4.6)	8.7
Parietal lobe	42.3 (4.7)	9.5
Left	21.3 (2.4)	4.8
Right	20.9 (2.4)	4.7
Temporal lobe	46.5 (5.0)	10.4
Left	21.4 (2.3)	4.8
Right	25.2 (2.8)	5.7
Occipital lobe	53.7 (6.3)	12
Left	33.8 (4.0)	7.6
Right	25 (2.9)	5.6

*ICV* intracranial volume, *TBV* total brain volume, *TWM* total white matter, *TGM* total gray matter volume, *TCV* total cortical volume

^a^Percentage of TBV in relation to ICV and all other volumes to TBV

### Interlobar correlations

**Fig. 3:**
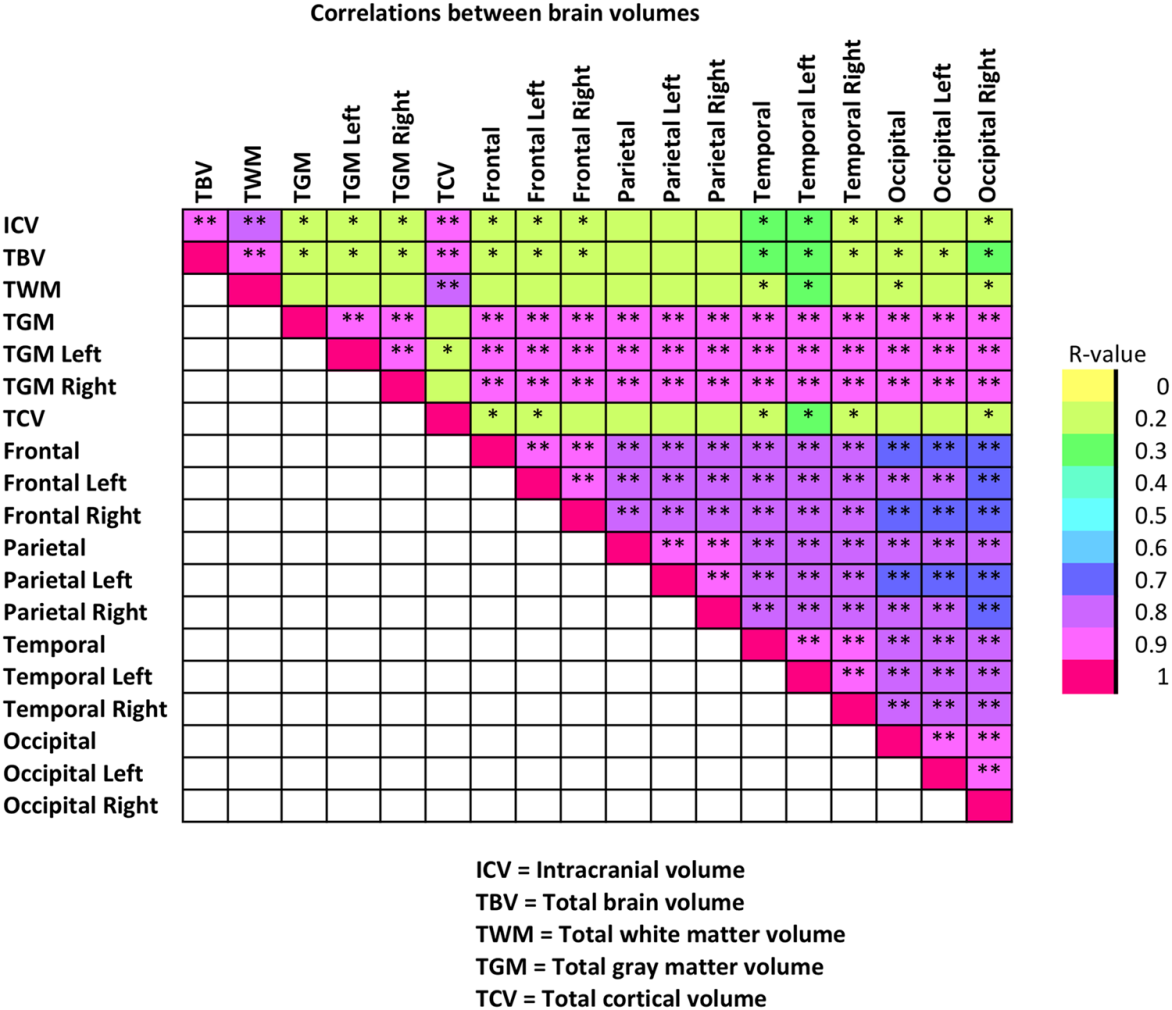
Intercorrelations of brain volumes. Colors indicate the value of the correlation coefficient and the strength of the relationship between brain volumes. Asterisks designate the statistical significance level (*p < 0.05; **p < 0.001)

Correlation analysis revealed that ICV correlated strongly with all the major tissue compartment volumes (TBV, TWM, TCV), which were also strongly correlated with each other (Fig. 3). Strong correlations between TGM and the following structures were also seen: right and left TGM; total, left and right volumes of frontal, parietal, temporal and occipital lobes. All lobar volumes were strongly correlated with each other. Interestingly, the strongest correlations in the analysis were between the temporal regions and the other lobes. TWM showed poorer correlation with frontal and parietal lobes and TCV with parietal lobe (Fig. 3).

### Correlations between infant growth metrics and brain volumes

With absolute volumes, head circumference was expectedly positively correlated with ICV, TBV, TWM and TCV (Table 3). Comparison with relative brain volumes showed a positive relationship between head circumference and frontal lobe volume and a negative relationship between head circumference and occipital lobe volume. No correlations were found between absolute or relative brain volumes and birth weight or length. After controlling for sex and age (counted from due date), all these findings persisted, and additionally head circumference was positively associated with absolute frontal lobe volume and negatively with total occipital lobe volume. Birth length was positively associated with absolute TGM (Table 3).

**Table 3:** Comparison of absolute and relative brain volumes and infant growth markers

Brain structures	Head circumference				Birth weight				Birth length			
	Absolute		Relative		Absolute		Relative		Absolute		Relative	
	*R*	*p*	*R*	*p*	*R*	*p*	*R*	*p*	*R*	*p*	*R*	*p*
ICV	0.319	0.008*			–	–			–	–		
TBV	0.319	0.009*			–	–			–	–		
TWM	0.340	0.005*	–	–	–	–	–	–	0.269	0.029*	–	–
TGM	–	–	–	–	–	–	–	–	–	–	–	–
Left	–	–	–	–	–	–	–	–	–	–	–	–
Right	–	–	–	–	–	–	–	–	–	–	–	–
TCV	0.289	0.019*	–	–	–	–	–	–	–	–	–	–
Frontal	0.262	0.039*	0.316	0.010*	–	–	–	–	–	–	–	-
Left	0.254	0.033*	0.289	0.018*	–	–	–	–	–	–	–	–
Right	–	–	0.312	0.011*	–	–	–	–	–	–	–	–
Parietal	–	–	–	–	–	–	–	–	–	–	–	–
Left	–	–	–	–	–	–	–	–	–	–	–	–
Right	–	–	–	–	–	–	–	–	–	–	–	–
Temporal	–	–	–	–	–	–	–	–	–	–	–	–
Left	–	–	–	–	–	–	–	–	–	–	–	–
Right	–	–	–	–	–	–	–	–	–	–	–	–
Occipital	–	–	− 0.330	0.007*	–	–	–	–	–	–	–	–
Left	–	–	− 0.272	0.027*	–	–	–	–	–	–	–	–
Right	–	–	− 0.329	0.007*	–	–	–	–	–	–	–	–

Pearson used for normally distributed values and Spearman for non-normally distributed values. Only statistically significant (*p* < 0.05) results are listed and marked with an asterisk. Bonferroni correction for multiple comparisons corresponding to *p* < 0.0058. *p* values meeting the significance criteria are bolded

*ICV* intracranial volume, *TBV* total brain volume, *TWM* total white matter, *TGM* total gray matter volume, *TCV* total cortical volume, *R* correlation coefficient, – no association/ non-significant

### Effects of neonate age on brain volumes

**Fig. 4:**
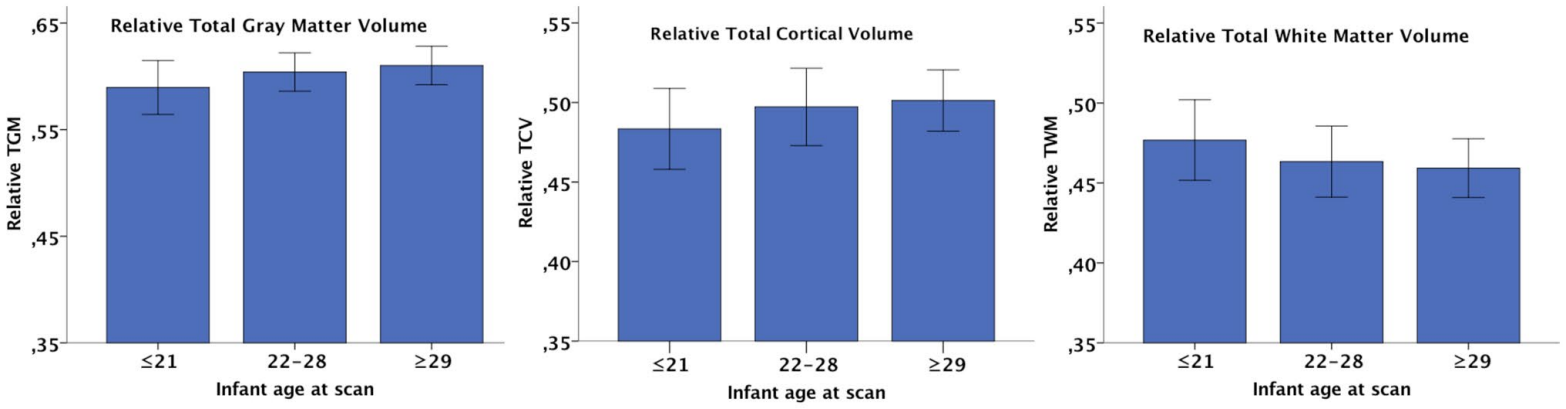
Relative total volumes and age at scan in days counted from estimated due date (*p* < 0.001). **a** Relative total gray matter volume; **b** relative total cortical volume; **c** relative total white matter volume. The bars represent standard deviations

No significant correlations were observed between the two calculated ages at scan and absolute total or lobar brain volumes in the study group as a whole (Table 4), but after controlling for sex, moderate positive correlations were seen between age counted from the estimated due date and TCV as well as total and right parietal lobe volumes. The correlations, separately for the subgroups of males and females, are presented in Table 4. The same analyses with relative brain volumes in the whole group showed a positive correlation between age and TGM as well as TCV and a negative correlation with TWM, regardless of if counting from the estimated due date or the actual birth date (Fig. 4). Table 4 provides a detailed report on the correlations.

**Table 4:** Correlations between absolute and relative brain volumes and infant age at scan counted from estimated due date

Cerebral structures	Absolute volumes			Relative volumes		
	All (*N* = 68)	Males (*N* = 31)	Females (*N* = 37)	All (*N* = 68)	Males (*N* = 31)	Females (*N* = 37)
	*p* (R)	*p* (R)	*p* (R)	*p* (R)	*p* (R)	*p* (R)
ICV	–	0.002* (0.540)	–			
TBV	–	0.001* (0.552)	–			
TWM	–	–	–	–	< 0.001* (− 0.637)	0.005*(− 0.454)
TGM	–	–	–	< 0.001* (0.605)	< 0.001* (0.650)	0.001* (0.538)
Left	–	–	–	< 0.001* (0.576)	< 0.001* (0.633)	0.001* (0.516)
Right	–	–	–	< 0.001* (0.480)	0.001* (0.578)	–
TCV	–	< 0.001* (0.645)	–	< 0.001* (0.559)	< 0.001* (0.682)	0.006* (0.446)
Frontal lobe	–	–	–	–	–	0.002* (− 0.486)
Left	–	–	–	–	–	0.004* (− 0.465)
Right	–	–	–	–	–	0.004* (− 0.461)
Parietal lobe	–	–	–	–	–	–
Left	–	–	–	–	–	–
Right	–	–	–	–	–	–
Temporal lobe	–	–	–	–	–	–
Left	–	–	–	–	–	–
Right	–	–	–	–	–	–
Occipital lobe	–	–	–	–	–	0.030* (0.357)
Left	–	–	–	–	–	0.017* (0.390)
Right	–	–	–	–	–	–

Pearson has been used for normally distributed values and Spearman for non-normally distributed values. Only statistically significant (*p* < 0.05) results are listed and marked with an asterisk. Bonferroni correction for multiple comparisons corresponding to *p* < 0.0028. *p* values meeting the significance criteria are bolded

*DD* age counted from estimated due date, *ICV* intracranial volume, *TBV* total brain volume, *TGM* total gray matter volume, *TCV* total cortical volume, – no correlation/non-significant, *ICV* intracranial volume, *TBV* total brain volume, *TWM* total white matter, *TGM* total gray matter volume, *TCV* total cortical volume

### Sex differences in brain volumes

A significant difference was found between the sexes in the absolute total volumes of the frontal and temporal lobes, so that in these structures, males had greater volumes (Table 5). A similar difference was seen in the right hemisphere and also in TGM. For the left hemisphere, this effect was only seen in the temporal lobe (Table 5). After correcting TGM with TVB and the lobar volumes with TGM, the results revealed a significant sex difference in TGM, the total volume of the parietal lobe, the volume of the right temporal lobe and the left parietal lobe so that females had greater relative volumes in these structures than males. No results survived the correction for multiple comparisons (Bonferroni, *p* < 0.006) (Table 5).

**Table 5:** Sex differences in mean absolute and relative brain volumes

Cerebral structures	Mean absolute volumes				Mean relative volumes			
	Males (*n* = 31), cm^3^ (SD)	Females (*n* = 37), cm^3^ (SD)	*t*	*p*	Males (*n* = 31)	Females (*n* = 37)	*t*	*p*
ICV	625.7 (60.7)	608.2 (55.2)		–				
TBV	450.8 (43.6)	442.6 (40.3)		–				
TWM	211.1 (18.7)	205.3 (18.0)		–	0.47	0.46		–
TGM	240.6 (26.2)	237. 4 (23.8)		–	0.60	0.61	− 2.26	0.027*
Left	122.3 (14.9)	116.6 (9.2)		–	0.27	0.27		–
Right	122.7 (15.0)	116.4 (9.0)	2.067	0.044*	0.27	0.27		–
TCV	222.0 (25.0)	219.8 (22.6)		–	0.49	0.50		–
Frontal lobe	80.8 (10.9)	76.1 (6.8)	2.107	0.040*	0.33	0.33		–
Left	40.3 (5.6)	37.9 (3.3)		–	0.17	0.16		–
Right	40.5 (5.3)	38.2 (3.5)	2.094	0.041*	0.17	0.16		–
Parietal lobe	43.4 (5.5)	41.3 (3.7)		–	0.18	0.18	− 2.373	0.021*
Left	21.9 (2.8)	20.9 (2.0)		–	0.09	0.09	− 2.443	0.017*
Right	21.6 (2.8)	20.4 (1.9)		–	0.09	0.09		–
Temporal lobe	47.9 (5.8)	45.4 (3.9)	2.098	0.041*	0.19	0.20		–
Left	22.0 (2.7)	20.9 (1.8)	2.02	0.049*	0.09	0.09		–
Right	25.9 (3.2)	24.5 (2.2)	2.09	0.042*	0.11	0.11	− 2.367	0.021*
Occipital lobe	54.8 (7.6)	52.7 (4.8)		–	0.23	0.22		–
Left	34.3 (4.7)	33.3 (3.2)		–	0.14	0.14		–
Right	25.7 (3.6)	24.5 (2.2)		–	0.11	0.11		–

Relative total volumes of grey and white matter were derived from relating absolute volumes to TBV and lobar absolute volumes to TGM. Independent samples *t* test was used with normally distributed values and Mann–Whitney *U* test with non-normally distributed values. Only statistically significant (*p* < 0.05) results are listed and marked with an asterisk

*ICV* intracranial volume, *TBV* total brain volume, *TWM* total white matter, *TGM* total gray matter volume, *TCV* total cortical volume, – no association/ non-significant

### Asymmetry

**Fig. 5:**
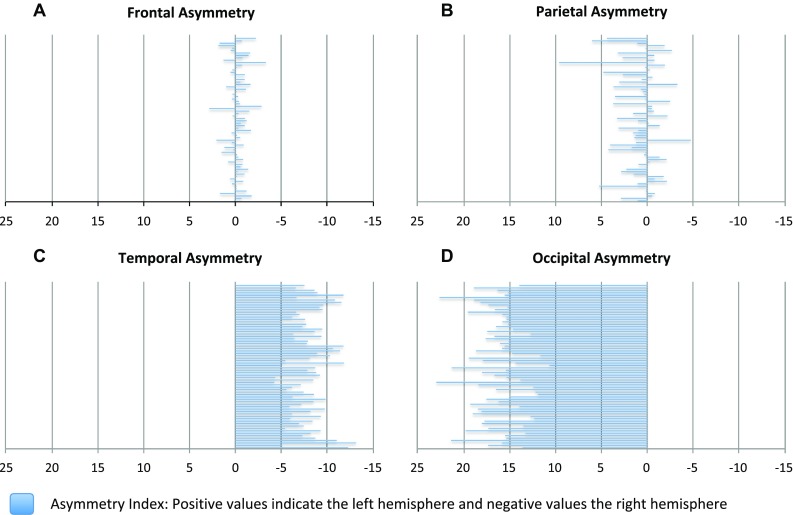
Lobar asymmetry retrieved from subtraction of the right side from the left and relating the result to the whole volume of the lobe. The negative values represent the right hemisphere and the positive values the left hemisphere. **a** Frontal lobe (result non-significant); **b** parietal lobe (*p* < 0.003); **c** temporal lobe (*p* < 0.0001); **d** occipital lobe (*p* < 0.0001)

The absolute volume of the left parietal lobe was 0.9% larger than the right in the whole group (*p* = 0.003). The right temporal and the left occipital lobes were significantly larger (8% and 16%) than the other side in the whole group (*p* < 0.0001) (Fig. 5). Asymmetry patterns were found to be similar in both sexes. All these findings persisted when corrected with TGM volume (Table 6). Regression analysis with sex as a fixed factor, and age as an independent factor revealed that age was not associated with the degree of asymmetry.

**Table 6:** Cerebral asymmetry

Brain volume	Left mean (SD)	Right mean (SD)	AI, L > R %	*t*	*p*	*p* (REL)
TGM (cm^3^)	119.2 (12.4)	119.2 (12.4)	− 0.038	− 0.43	–	–
Frontal (cm^3^)	39.0 (4.6)	39.2 (4.6)	− 0.263	− 1.93	–	–
Parietal (cm^3^)	21.3 (2.4)	21.0 (2.4)	0.899	3.05	0.003*	0.003*
Temporal (cm^3^)	21.4 (2.3)	25.2 (2.8)	− 8.131	− 32.93	< 0.0001*	< 0.0001*
Occipital (cm^3^)	33.8 (4.0)	25.0 (2.9)	16.282	51.91	< 0.0001*	< 0.0001*

Comparison between left and right hemisphere. *p* values are derived from one sample *t* test. Significance level (*p* < 0.05) is marked by an asterisk. Bonferroni correction for multiple comparisons corresponding to *p* < 0.01. *p* values meeting the significance criteria are bolded

*AI* Asymmetry Index, *TBV* total brain volume, *TGM* total gray matter volume, *p(REL)* TGM controlled with TBV and lobar volumes with TGM; – non-significant

## Discussion

### Interlobar correlations

Our correlation analysis revealed that although all lobar volumes were highly correlated with each other, the temporal regions had the highest correlation with all other lobar regions while the occipital region had the weakest relationships with the right frontal and the left parietal lobes. While the anatomical structure of the brain can change as a result of functional specialization (Lerch et al. 2006), one could expect that the way the anatomical regions are correlated might reflect the functional interaction between these brain structures, and that a change in the correlations might serve as a developmental marker in addition to asymmetry. The strong connection of other brain regions to the temporal lobes is logical as they execute many important sensory information processing and cognitive functions. However, we acknowledge that correlations are a statistical construct that reflect connectivity information to a very limited extent (Irimia and Van Horn 2013) and thus by reporting them did not aim to describe connectomics. It is clear that to draw further conclusions on the interrelationships between structures and function would require the use of additional data sources.

### Brain volume relations with infant body size

Concerning neonatal growth markers, including birth weight, birth length and head circumference, both sex groups had similar results (Table 4). Of these infant metrics, greater head circumference was expectedly associated with larger absolute volumes of ICV, TBV, TWM, TCV, but also with larger absolute and relative volumes of frontal lobes, and smaller relative volumes of the occipital lobe. We found no correlation between birth weight or length and brain volumes, but after controlling for sex, a positive correlation was seen between birth length and TWM. The effect of body size should always be evaluated and, if necessary, acknowledged in the analyses, especially in special prenatal conditions that could influence growth (Walhovd et al. 2012), although our sample consisted of only healthy, term-born individuals.

### Brain volume relations with age

Age expectedly associated positively with the size of TGM and TCV and negatively with that of TWM in both sexes. In addition, in males, age predicted positively also the absolute volumes of ICV and TBV. As for the negative association between age and TWM in females, it has been reported before (Knickmeyer et al. 2008a, b), where the effect was seen during a follow-up of 24 months; yet, the majority of the evidence regarding the effect of age on both gray and white matter volume indicates the relationships to be positive (Bora et al. 2014; Holland et al. 2014; Peterson et al. 2000). Gray matter volume increases during the first years of life, while previous studies have argued that the growth of white matter is steadier, but likely extends into adulthood (Knickmeyer et al. 2008a, b). Our results support this by showing that white matter growth is slower compared to that of gray matter in the early stages of life.

In contrast to the previous findings, a recent large study (*n* = 756) (Knickmeyer et al. 2017) on the effects of demographic and obstetric factors on infant brain volumes, detected that infants born earlier had larger brain volumes than later born infants (gestational age at birth ranging from 27 to 46 weeks). Importantly though, this finding was only apparent when the volumes were adjusted to gestational age at MRI and birth weight. The authors speculated this to be the result of accelerated brain growth happening in the premature brain after birth. Nevertheless, infants born earlier had some smaller volumes in the visual association cortex, primary auditory, and auditory association cortex, which is in line with previous studies on premature infants (Knickmeyer et al. 2017). This latter finding of shorter gestation being associated with smaller brain volumes, together with our results, in which greater age predicted larger brain volumes during the first weeks of postnatal life of term-born infants, support the earlier reports of the positive connection between brain volumes and longer gestation.

### Brain volume relations with sex

Partly in conflict with previous evidence (Dean et al. 2018; Gilmore et al. 2007; Knickmeyer et al. 2017), we found no sex differences in ICV, TBV, TWM or TCV. However, neither did Gilmore when correcting for ICV (Gilmore et al. 2007). In our findings, TGM of the right hemisphere was greater in boys, but after controlling with TBV, the finding did not persist, thus supporting the findings of Munakata et al. on late-preterm and term infants showing no relationship between gray matter volume and sex (Munakata et al. 2013). Hence, the sex-related dimorphic volumetric differences might not be seen in the total volumes, but rather more regionally, balancing each other out so that the total volumes stay relatively equal. The diversion in results might also be the product of diverse sample sizes (Dean et al. 2018; Gilmore et al. 2007; Munakata et al. 2013).

As in previous research (Knickmeyer et al. 2008a, b; van Soelen et al. 2010), our results support the notion that male brains have greater absolute lobar volumes than females, specifically, the total and right side volumes of the frontal, temporal and parietal regions. Interestingly, females presented relatively larger volumes of TGM, of the total and left parietal lobes and of the right temporal lobe. These results align with the result of a previous study, done on children and young adults, showing greater gray matter to white matter ratio in females (Koolschijn and Crone 2013). However, the sex differences in our findings were modest and might not be of biological significance. Nevertheless, combining existing findings with our findings on the larger particular absolute volumes in males as well as the larger specific relative volumes in females provides support for theorizing that possible sex differences in the infant brain are more intricate and regional. However, the information concerning regional sexual dimorphism is still scarce and further definitive conclusions cannot be made.

### Brain asymmetry

We detected similar lobar asymmetry in both sexes: rightward in the temporal lobe and leftward in parietal and occipital lobes. This right-larger-than-left asymmetry in temporal lobe is strongly consistent with previous research on early asymmetry (Dean et al. 2018; Mark et al. 1999; Matsuzawa et al. 2001; Rajagopalan et al. 2011; Tanaka et al. 2013) and although it has been hypothesized to be associated with the development of speech and language, its presence in the “prespeech population” of newborns defends the theory that brain asymmetry exists already in the infant brain before the development of any communication skills. However, discrepant evidence exists also on hemispheric differences that contradict with the rightward asymmetry findings. Gilmore at al. found left-larger-than-right hemispheric asymmetry in infants and also a laterality difference that was greater in females: the left occipital and prefrontal regions being larger than the right, although the result for the prefrontal area was non-significant and they reported findings on absolute volumes only (Gilmore et al. 2007). While our results do not concur with these hemispheric asymmetry findings, we did see a coinciding pattern of the left occipital lobe being larger than the right.

In this study population, age was not associated with asymmetry. Nevertheless, different hemispheric growth patterns might appear later, as it has been seen in longitudinal settings in older children (Matsuzawa et al. 2001; Tanaka et al. 2013). This may be a consequence of the different hierarchical functions of the brain networks: later maturing parts contain association areas that integrate input from different sensory sources (Tanaka et al. 2013). In conclusion, the detected similar asymmetries in parietal, temporal and occipital lobes in both sexes are highly accordant with previous findings and confirm the already existing observations on early asymmetry. These brain alterations possibly support overall development, yet more longitudinal research is needed on the matter.

### Strengths and limitations

A key strength of the present study was that the individuals were scanned soon after birth to prevent the confounding effects of postnatal factors. The age variance was nevertheless broad enough to justify the age-volume correlation analyses. In addition, only full-term infants were included minimizing the complex and possibly confounding effects of prematurity and other pregnancy complications. The same scanner and scanning protocol were used for all individuals. The generalizability of these results is subject to certain limitations. For instance, lobar volumes are a gross, yet robust, parameter to evaluate brain growth let alone brain development, as volumetric differences do not reveal directly the functional development of the nervous system. Additionally, the data included only full-term Finnish infants and may not be generalizable to other infant groups or nationalities. As the study is cross-sectional, the possible functional correlates remain to be studied in the future. Finally, the narrow age range inhibited further investigation of potential sex differences in brain growth patterns.

## Conclusion

In this study, we examined lobar asymmetry and the relations of brain volumes to infant age (2–5 weeks of age) and sex. We discovered that lobar asymmetry was present in the infant brain and similar in both sexes; however, the degree of asymmetry had no association with infant age. As expected, age predicted positively the volume of gray matter and negatively that of the white matter reflecting the different growth rates and patterns of both brain matters. We also found some regional gray matter volume differences between sexes suggesting that sexual dimorphism in brain development may be seen already at this stage, but only in some parts of the brain. This research extends previous information on early brain development.
